# Hepatitis B virus (HBV) receptors: Deficiency in tumor results in scant HBV infection and overexpression in peritumor leads to higher recurrence risk

**DOI:** 10.18632/oncotarget.5518

**Published:** 2015-10-20

**Authors:** Ying-Ying Jing, Wen-Ting Liu, Shi-Wei Guo, Fei Ye, Qing-Min Fan, Guo-Feng Yu, Dan-Dan Yu, Lu Gao, Kai Sun, Zhi-Peng Han, Rong Li, Yang Yang, Qiu-Dong Zhao, Meng-Chao Wu, Hong-Yang Wang, Li-Xin Wei

**Affiliations:** ^1^ Tumor Immunology and Gene Therapy Center, Eastern Hepatobiliary Surgery Hospital, The Second Military Medical University, Shanghai, China; ^2^ Central Laboratory, Ren Ji Hospital, School of Medicine, Shanghai Jiao Tong University, Shanghai, China; ^3^ International Cooperation Laboratory on Signal Transduction, Eastern Hepatobiliary Surgery Institute/Hospital, The Second Military Medical University, Shanghai, China

**Keywords:** hepatitis B virus receptor, inflammation, hepatic progenitor cells, recurrence

## Abstract

Hepatitis B virus (HBV) infection is a risk factor for hepatocarcinogenesis and recurrence. Here, we sought to characterize intratumoral and peritumoral expression of HBsAg and its specific receptors in HBsAg-positive hepatocellular carcinoma (HCC) patients and further examined their correlation with the recurrence-free survival (RFS). HCC tissue and adjacent normal tissue specimens were acquired from HBsAg-positive patients. The presence of HBsAg and receptors, as well as hepatic progenitor cells (HPCs) were detected by tissue microassay and immunohistochemistry. Necroinflammatory activity was evaluated by HE staining. The mean IOD of HBsAg and HBV DNA in the intratumoral tissues was markedly lower than that in the peritumoral tissues (*P* < 0.001). Pearson correlation analysis further showed a significant correlation between the expression of HBsAg and NTCP (*r* = 0.461, *P* < 0.001) or ASGPR (*r* = 0.506, *P* < 0.001) in peritumoral tissues. And the peritumoral HBsAg and receptors presented a positive association with necroinflammatory activity (*P* < 0.05). Inflammation induced by HBV infection presented a positive association with HPCs activation (*P* < 0.05). Additionally, due to lack of HBV receptors, HPCs was not preferentially infected with HBV, but activated HPCs had a significant correlation with HBsAg expression in peritumoral tissues, and the peritumoral HPCs activation was associated with RFS of HCC patients, therefore, the overexpression of HBsAg and receptors in peritumor were also with higher recurrence risk (*P* < 0.05). In conclusion, lack of HBV receptors resulted in scant HBV infection in tumor cells, and overexpression of HBsAg and receptors in peritumor was strongly associated with higher recurrence risk in HCC patients.

## INTRODUCTION

Hepatocellular carcinoma (HCC) is the third leading cause of cancer-related mortality and the fifth most common form of cancer globally [1]. Chronic hepatitis B virus (HBV) infection, which affects approximately 250 million people worldwide, substantially elevates HCC risk [2]. Long term persistence of hepatitis B surface antigen (HBsAg) in chronic hepatitis B patients has been considered a risk factor for HCC development [3]. More recently, high levels of HBsAg were found to be associated with poorer overall survival (OS) and recurrence free survival (RFS) of HBsAg-positive postoperative HCC patients with HBV DNA levels < 2000 IU/mL [4]. Currently, scant information is available on intratumoral and peritumoral HBsAg expression in HBsAg-positive HCC patients and its correlation with patient outcome.

HBV gains entry into hepatocytes via specific viral receptors sodium taurocholate cotransporting polypeptide (NTCP) and the hepatic asialoglycoprotein receptor (ASGPR) [5]. Little information is available on the role of NTCP in HCC. An early study indicated that NTCP expression was markedly reduced in most HCCs though its significance remained unknown [6]. There are also very limited studies on ASGPR expression in human HCC tissues. In a tissue microarray study, among eleven matched-pair sets, the normal liver tissues showed higher, but statistically insignificant, ASGPR expression compared with HCC tissues [7]. A more recent study of 62 HCC patients showed that high ASGPR expression in HCC tissues was associated with lower cumulative survival of these patients [8]. These studies, however, were undertaken without taking into consideration the HBsAg status in the HCC patients.

In the current study, we sought to characterize intratumoral and peritumoral expression of HBsAg and its specific receptors NTCP and ASGPR in 115 HBsAg-positive HCC patients by tissue microarray and immunohistochemistry and further examined their correlation with the RFS of these patients.

## RESULTS

### Patient demographic and baseline characteristics

**Figure 1:**
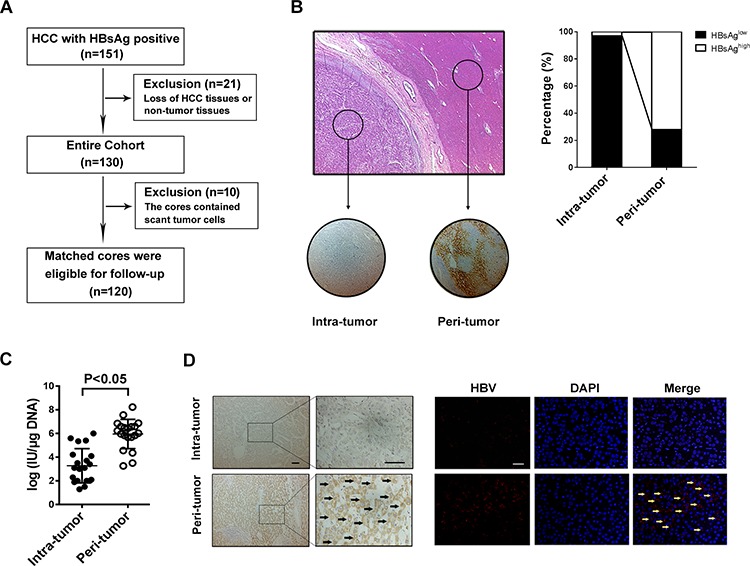
HBV infection in HCC tissues is lower than that in the surrounding liver tissue **A.** Patient demographic and baseline characteristics. **B.** Tissue microarray section contains tumor tissue and paired-surrounding tissue identified by H&E staining, and immunostaining with anti-HBsAg antibody shows that HBsAg expression was mainly localized to the cytoplasm and membrane (right); Of the 120 pairs samples, the percentage of HBsAg-high staining in intra-tumor is less than in peri-tumor (left). **C.** RT-PCR is used to detecte HBV DNA load fresh HCC tissue and matched surrounding tissue, and the results show that the median DNA level is much lower in tumor tissue than in peri-tumor tissue (*P* < 0.001). **D.** The specific whole length HBV DNA is used as probe to perform FISH on paraffin tissue samples, and the surrounding normal liver cells present positive signals for HBV DNA, but most tumor cells don't have FISH signal no matter with Diaminobenzidine (left) or immunofluorescence staining (right).

The study flow chart is shown in Figure 1A. 151 HBsAg-positive HCC patients underwent curative resection during 1997–2007. 21 patients were excluded because of loss of tissue specimens, and 10 patients were excluded because the cores contained scant tumor cells. 120 matched HCC tissue and adjacent normal tissue specimens were eligible for further screening until study closure in 2012, which were all followed the REMARK guidelines. They were from 97 (80.8%) males and 23 (19.2%) females with a mean age of 53.02 ± 11.62 years (range: 11–89 years). The demographic and baseline characteristics of the study subjects are shown in Supplementary Table S1. The mean tumor size was 5.9 ± 3.2 cm. The majority had TNM stage I or II HCC (91.7%) and showed grade I or II tumor differentiation (70%). Lymph node metastasis was present in less than 9% of the patients.

### HBsAg-positive HCC patients exhibit significantly higher HBsAg expression and HBV DNA load in peritumoral tissues than intratumoral tissues

Tissue microarray and immunohistochemistry revealed localization of HBsAg mainly in the cytoplasm and membrane (Figure 1B), and high HBsAg expression was demonstrated in 72% of the peritumoral tissue specimens, but was seen in only 3% of the intratumoral tissue specimens. Our fluorescence quantitative real-time PCR further revealed a significantly higher HBV DNA load in 20 additional fresh peritumoral tissue specimens (5.96 ± 0.28 log IU/μg) than the paired intratumoral tissue specimens (3.28 ± 0.32 log IU/μg) (*p* < 0.001) (Figure 1C). FISH demonstrated positive HBV DNA signals mostly in the peritumoral tissues while most tumor cells did not exhibit a FISH signal (Figure 1D). These findings indicated that compared to the intratumoral tissues, the peritumoral tissues had significantly greater HBsAg levels and higher HBV DNA load in HBsAg-positive HCC patients.

### The distribution of NTCP and ASGPR was consistent with HBsAg in HCC tissues

**Figure 2:**
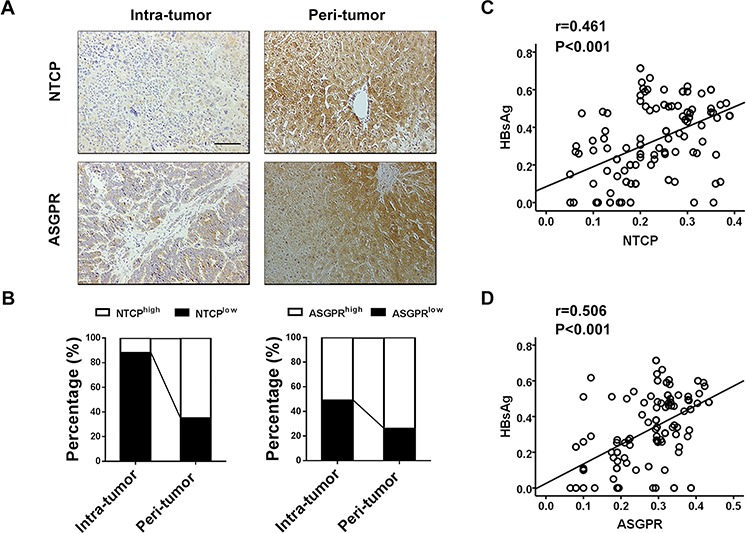
HBsAg expression significantly correlates with NTCP and ASGPR expression in peritumoral tissues of HBsAg-positive HCC patients **A.** Immunostaining for NTCP and ASGPR reveals a positive membranes pattern in peri-tumor cells, whereas less expressions in tumor cells. **B.** Compared with corresponding intra-tumor tissues, the percentages of NTCP and ASGPR high expression are both significantly increased in the peri-tumor tissues. **C.** and **D.** Scatterplots with fitting line show positive correlation between HBsAg and NTCP or ASGPR expression (IOD) in peri-tumor tissue, Pearson correlation provides correlation coefficient (*r*) and *P* value.

HBV gains entry into human hepatocytes via specific viral receptors, such as NTCP and ASGPR [9, 10], and we were interested in examining the expression of NTCP and ASGPR in intratumoral and peritumoral tissues from HBsAg-positive HCC patients. Immunostaining revealed a strongly positive intracytoplasmic and membranous pattern of NTCP and ASGPR in peritumoral cells, but a weakly positive expression in intratumoral cells (Figure 2A). Moreover, 78 (65%) and 88 (73%) peritumoral specimens exhibited high NTCP expression and high ASGPR expression, respectively, while 42 (35%) and 32 (27%) peritumoral specimens showed low NTCP expression and low ASGPR expression, respectively (Figure 2B). Furthermore, we observed that the surrounding tumor tissues with different HBsAg expression have different levels of NTCP (*p* < 0.001) and ASGPR (*p* < 0.001, Supplementary Figure 1A). Some cases of peri-tumor with intensive HBsAg staining showing high expression of NTCP or ASGPR staining as showing in Supplementary Figure 1B. Pearson correlation analysis further showed a significant correlation between HBsAg expression and NTCP expression (*r* = 0.461, *p* < 0.001, Figure 2C) and between HBsAg expression and ASGPR expression in peritumoral tissues (*r* = 0.506, *p* < 0.001, Figure 2D). Together, these findings demonstrated that HBsAg expression significantly correlated with NTCP and ASGPR expression in peritumoral tissues of HBsAg-positive HCC patients and higher HBV receptor levels were associated with higher HBsAg expression in the peritumoral tissue.

### Peritumoral HBsAg, NTCP, and ASGPR expression positively correlates with necroinflammatory activity in HCC tissues

Compared to patients who had low peritumoral HBsAg expression, significantly more patients with high peritumoral HBsAg expression had tumor size ≥ 5 cm (*p* = 0.008), microvascular invasion (*p* < 0.001), Edmondson grade I and II (*p* = 0.026), and high level of AFP (*p* = 0.015) (Supplementary Table S2). In addition, compared to patients who had low peritumoral NTCP or ASGPR expression, significantly more patients with high peritumoral NTCP or ASGPR expression had tumor size ≥ 5 cm (NTCP: *p* = 0.007; ASGPR: *p* = 0.005) and microvascular invasion (NTCP: *p* < 0.001; ASGPR: *p* < 0.001) (Supplementary Table S2). Spearman correlation analysis further showed that high HBsAg, NTCP or ASGPR expression was positively associated with tumor size (HBsAg: *r* = 0.240, *p* = 0.008; NTCP: *r* = 0.247, *p* = 0.007; ASGPR: *r* = 0.255, *p* = 0.005) and microvascular invasion (HBsAg: *r* = 0.589, *p* < 0.001; NTCP: *r* = 0.412, *p* < 0.001; ASGPR: *r* = 0.377, *p* < 0.001).

**Figure 3:**
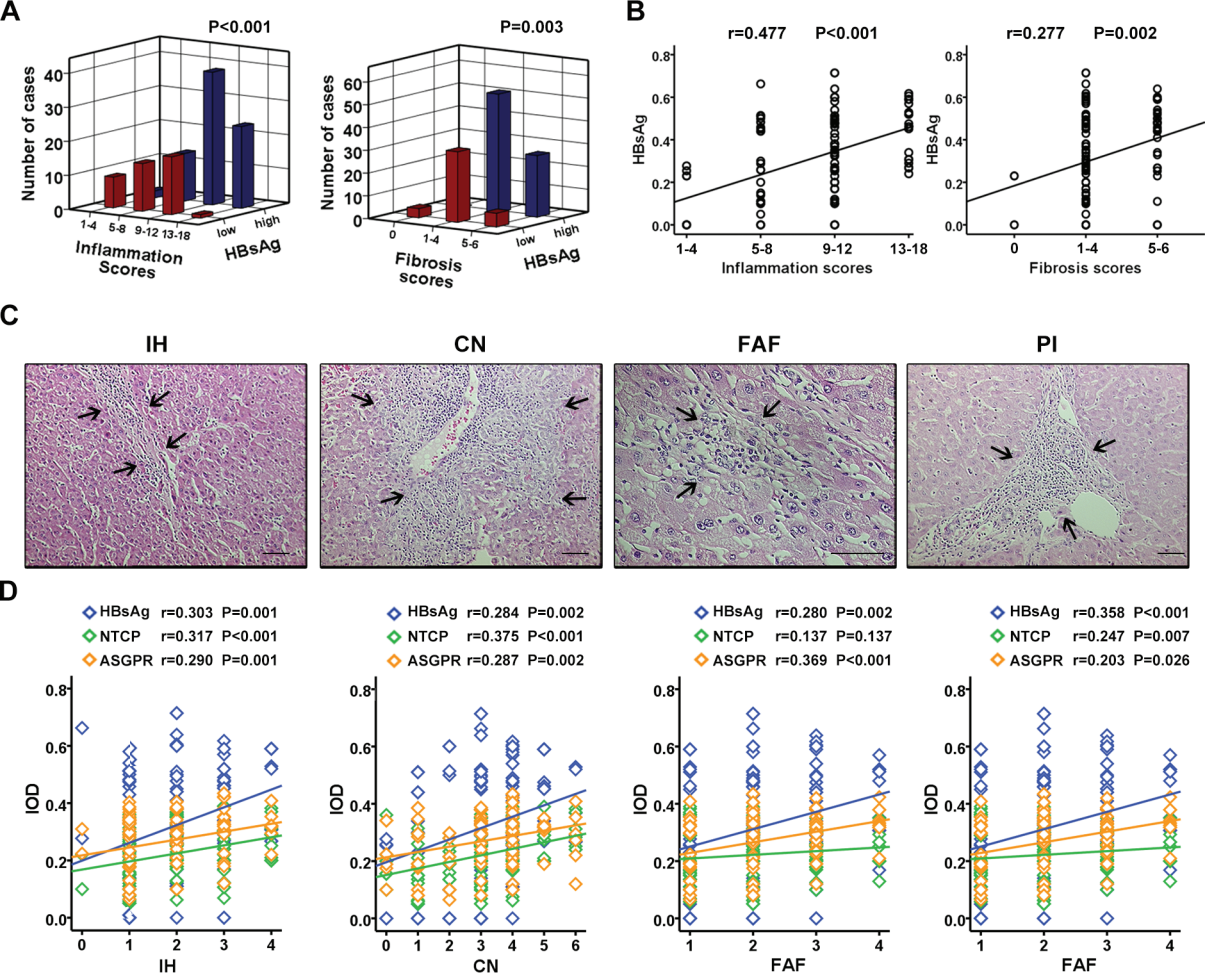
Peritumoral HBsAg, NTCP, and ASGPR expression positively correlates with necroinflammatory activity in HCC tissues **A.** HCC surrounding tissues with different HBsAg expression has different scores of inflammation grade (*P* < 0.001) and fibrosis stage (*P* = 0.003). **B.** Scatterplot with fitting line shows HBsAg expression positively correlates with inflammation scores (*r* = 0.477, *P* < 0.001), as well as fibrosis scores (*r* = 0.277, *P* = 0.002). **C.** The histomorphology of inflammation subtypes including IH, CN, FAF and PI by H&E staining. **D.** Scatterplot with fitting line shows the association of HBsAg expression, as wll as receptors expressions, with the four inflammation subtypes respectively. The correlation coefficient (*r*) and *P* value are showed in figure.

Inflammation is a noticeable feature of chronic hepatitis B infection-induced liver injury. Pearson correlation analysis showed that HBsAg expression positively correlated with inflammation grade (*r* = 0.477, *p* < 0.001) and fibrosis scores (*r* = 0.277, *p* = 0.002) (Figure 3A, 3B). There was also a significant positive correlation between HBsAg, NTCP, or ASGPR expression with interface hepatitis or confluent necrosis (Figure 3C, 3D, left two panels). A significant positive correlation was noticed between HBsAg or ASGPR expression and focal lytic necrosis/apoptosis/focal inflammation (Figure 3C, 3D, second right panel) while HBsAg or NTCP expression exhibited a significant positive correlation with portal inflammation (Figure 3C, 3D, the right panel). These findings demonstrated that peritumoral expression of HBsAg, NTCP, and ASGPR positively correlated with necroinflammatory activity in HCC tissues.

### Scant expression of HBsAg and receptors on HPCs activated in inflammation microenvironment

**Figure 4:**
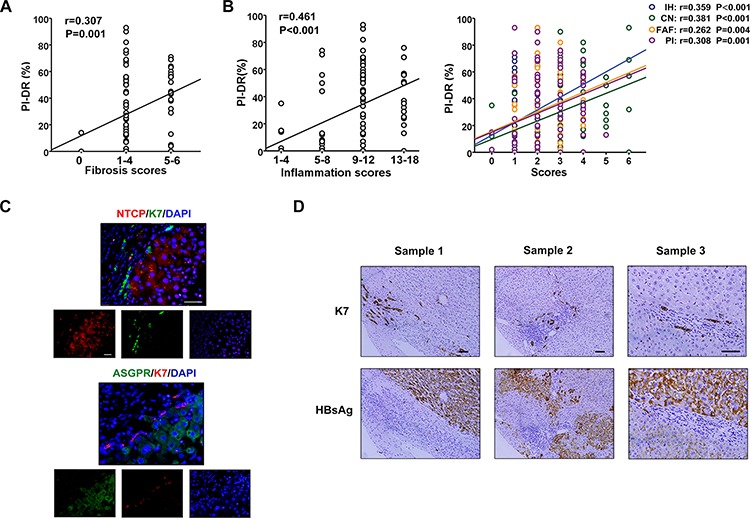
Scant expression of HBsAg and receptors on HPCs activated in inflammation microenvironment **A.** PI-DR was showed a positive association with the stage of fibrosis. **B.** There was a significant positive correlation between PI-DR with the grade of inflammation, as well as interface hepatitis or confluent necrosis, focal lytic necrosis/apoptosis/focal inflammation, portal inflammation. **C.** The double immunostaining shows that co-expression of K7 and HBV receptors (NTCP or ASGPR) in hepatocytes is rare virtually. **D.** Immunostaining in the serial section presented that HBsAg expressed mostly in mature hepatocytes, but not in portal area, especially K7 positive HPCs.

We have previously shown that the PI-DR in HCC tissues correlated with the number of isolated hepatic progenitor cells (HPCs) and intermediate hepatobiliary cells [11]. Here, PI-DR was showed a positive association with the grade of inflammation and the stage of fibrosis (Figure 4A, 4B), which was confirmed our previous study. We further selected peritumoral tissue specimens to examine the expression of K7 and HBV receptors. The immunofluorescent microscopy revealed scant co-expression of K7 and NTCP or ASGPR in HPCs (Figure 4C), and serial sectioning of the tissue specimens showed that HBsAg was expressed mostly in mature hepatocytes, and was mainly distributed in the central vein zone, not in the portal area, especially in K7 positive HPCs (Figure 4D). Immunostaining revealed co-expression of HBsAg with CYP3A4, a cytochrome enzyme in mature hepatocytes (Supplementary Figure S2A) and with MRP2, a polarization marker of hepatocytes [12] (Supplementary Figure S2B). In addition, NTCP or ASGPR was co-expressed with MRP2 (data not shown). These results indicated that HBsAg was not as highly expressed in HPCs with scant NTCP and ASGPR expression as in mature hepatocytes.

### Overexpression of peritumoral HBsAg, NTCP and ASGPR is associated with HPCs activation and predicted higher recurrence risk of HCC patients

**Figure 5:**
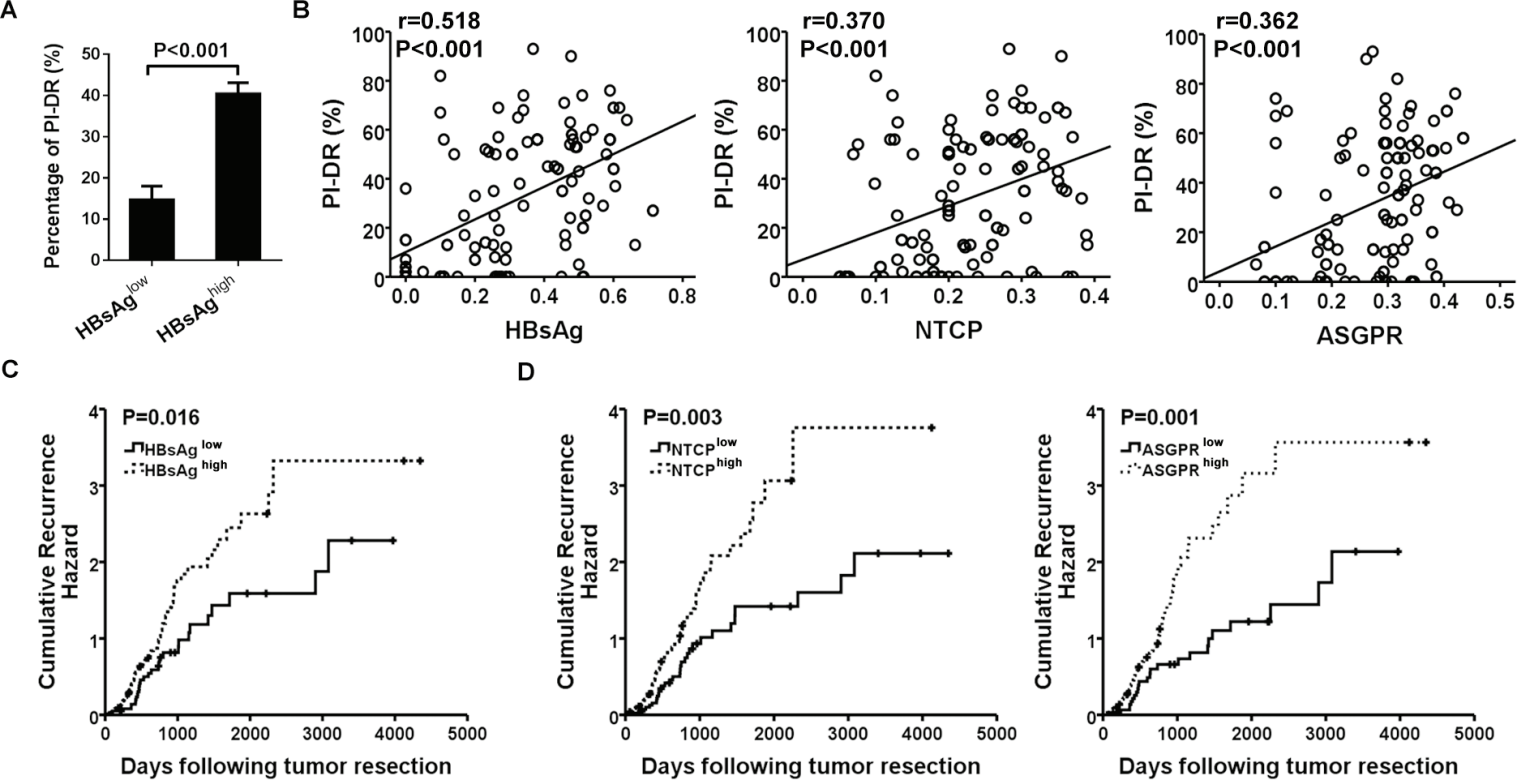
Overexpression of peritumoral HBsAg, NTCP and ASGPR is associated with HPCs activation and predicted higher recurrence risk of HCC patients **A.** Higher expression of HBsAg showed greater percentage of PI-DR in non-tumor sections. **B.** Expression of HBsAg and receptors in peri-tumor tissues showed a positive correlation with PI-DR, Pearson correlation provides correlation coefficient (*r*) and *P* value. **C.** and **D.** Cumulative recurrence hazard analysis showed that patients with high peritumoral HBsAg and receptors expression were more likely to have recurrence.

We further detected the relationship of HBV infection and HPCs activation, and the results showed that PI-DR was significantly higher in peritumoral tissues with overexpression of HBsAg than those with low HBsAg expression (*P* < 0.001) (Figure 5A), and Pearson correlation analysis revealed a significant positive correlation between HBsAg expression and the PI-DR. In addition, the receptors were also showed a close association with HPCs activation (Figure 5B). Increased PI-DR was confirmed to be associated with poor OS and RFS of patients, and our results showed that higher PI-DR could contribute to the risk of HCC recurrence (Supplementary Figure S3). Furthermore, our univariate analysis revealed that peritumoral HBsAg, NTCP and ASGPR expression was associated with RFS (*P* = 0.017 for HBsAg, *P* = 0.003 for NTCP and *P* = 0.002 for ASGPR) and OS (*P* = 0.005 for HBsAg, *P* = 0.017 for NTCP and *P* = 0.001 for ASGPR), and risk factors (tumor size and microvascular invasion) identified by univariate analysis were pooled into a multivariate Cox proportional hazards analysis (Supplementary Table S3). Moreover, in survival analyses within the curative resection group, patients with high peritumoral HBsAg expression were more likely to have higher cumulative recurrence hazard (Figure 5C) and poorer OS (Supplementary Figure S4A), and the patients with high peritumoral ASGPR or NTCP expression also had the similar recurrence risk (Figure 5D) and overall survival (Supplementary Figure S4B).

## DISCUSSION

Persistence of HBV infection has long been considered the most common cause of HCC, as well as HCC recurrence after its curative treatment [13, 14]. Recent evidence suggests that high HBsAg levels or high ASGPR expression in HCC tissues are associated with lower cumulative survival of HCC patients [4]. In the current study, we demonstrated that HBsAg-positive HCC patients exhibited significantly higher HBsAg expression in peritumoral tissues than intratumoral tissues, which significantly correlated with peritumoral NTCP and ASGPR expression. Importantly, we showed that high peritumoral HBsAg, NTCP and ASGPR expression was associated with high recurrence risk of HBsAg-positive HCC patients. To our knowledge, few studies have evaluated the prognostic significance of intratumoral and peritumoral expression of HBsAg and its specific receptors NTCP and ASGPR in HBsAg-positive HCC patients. Our analysis indicates that peritumoral expression of HBsAg, NTCP and ASGPR correlates with patient recurrence and may serve as useful biomarkers for patient outcomes.

Although an etiological association between HBV infection and HCC development has long been established, the molecular mechanism of hepatocarcinogenesis still remains poorly understood. Our current findings showed that HBsAg expression correlating with NTCP and ASGPR is consistent with the role of NTCP and ASGPR as specific HBV receptors. Another striking finding of the current study is high peritumoral HBsAg expression in HBsAg-positive HCC patients, which is consistent with the finding of a smaller study on 13 HBsAg-positive HCC tissues [15]. This high peritumoral HBsAg expression mirrors high peritumoral NTCP and ASGPR expression, suggesting reliance on the viral receptors by HBV to gain entry into hepatocytes.

Furthermore, we have also found that HBsAg is mostly expressed in mature hepatocytes. During HCC development, absence or decreased expression of HBsAg in tumor cells is a common phenomenon [16–18], but the exact mechanism is still not clear. Wang *et al*. attributed it to direct HBV integration in tumor cells [17]. Our findings offer a more plausible explanation that absence or decreased expression of HBsAg in tumor cells is due to low intratumoral expression of NTCP and ASGPR. NTCP expression in the indicated HCC cell lines and primary hepatocytes has also been detected by Yan et al in lab experiment, the results showed that the levels of NTCP mRNA in Huh-7 and HepG2 cells were about 10,000 times lower than that in primary human and Tupaia hepatocytes, which was consistent with our observation in clinical tissues [19]. However, how HBsAg, NTCP and ASGPR contribute to HCC development remains unknown. Our multivariate analysis indicates that high peritumoral expression of HBsAg, NTCP and ASGPR in HBsAg-positive HCC patients is associated with higher risk of tumor recurrence.

**Figure 6:**
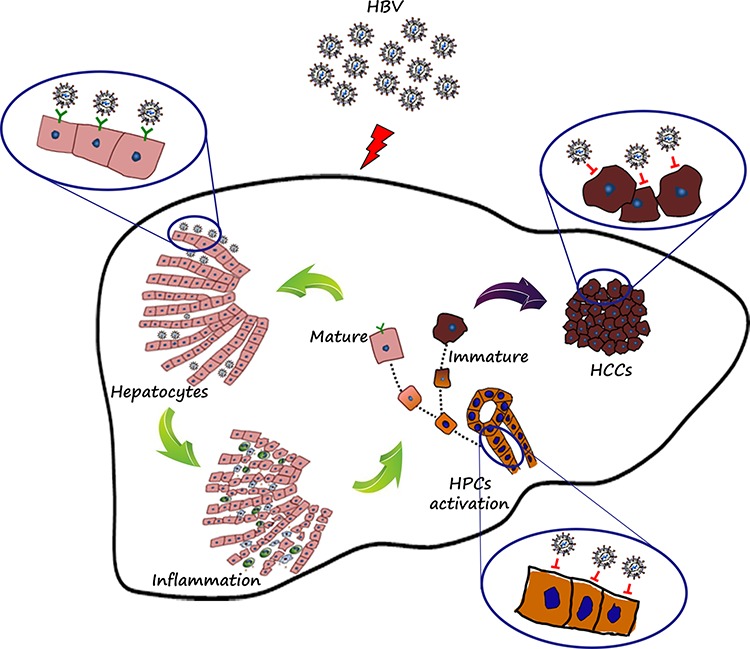
Diagrammatic drawing HBV receptors determined the fate of cells with or without HBV infection. Mature hepatocytes with HBV receptors (NTCP and ASGPR) were apt to HBV infection, persistent HBV infection resulted in prolonged and consistent inflammation, and in the inflammation microenvironment, HPCs were activated exactly, but not co-expressed with HBsAg, which mainly resulted from lack of HBV receptors. If the progenitor cells existing in the inflammation microenvironment continued to differentiate into hepatocytes, then the mature cells with polarization would be infected with HBV. Conversely, if the progenitor cells continued to maintain this undifferentiated state in the ongoing inflammation microenvironment, then the immature cells would be gathered and eventually deteriorate into tumors, which may further explain the phenomenon of lower HBV infection in tumor tissues.

We speculated that the association of high peritumoral expression of HBsAg, NTCP and ASGPR with the distal outcome of HBsAg-positive HCC patients may be due to the intimate association of HBV infection and inflammation. Previous studies have proved that chronic HBV infection leads to ongoing inflammation and continuous hepatocyte regeneration [20, 21]. Inflammation associated with chronic active hepatitis is a major contributor in hepatocarcinogenesis [22]. Repeated episodes of inflammation, apoptosis and hepatocyte regeneration also increase the risk of HCC recurrence [22]. Our Pearson correlation analysis has demonstrated a positive correlation of peritumoral HBsAg expression with inflammation grade and focal inflammation and portal inflammation. In the inflammatory microenvironment, HPCs, which lack HBV receptors, are activated. If the HPCs become differentiated into hepatocytes, they would be infected with HBV. Conversely, if they continued to maintain their undifferentiated state in the inflammatory microenvironment, they would be free of HBV infection, the mainly diagrammatic drawing was shown in Figure 6.

Substantial clinical evidence indicates that persistent HBV infection accelerates the progression of liver fibrosis, which is considered the most important risk factor for HCC development. Libbrecht *et al*. found that the number of HPCs correlated significantly with the severity of parenchymal inflammation [23]. We have previously shown that the PI-DR in HCC tissues correlated with the number of isolated HPCs and intermediate hepatobiliary cells [11]. The current study also revealed that the PI-DR was significantly higher in peritumoral tissues with high HBsAg expression than those with low HBsAg expression in HBsAg-positive HCC patients and showed a positive association with the grade of inflammation and the stage of fibrosis (Supplementary Figure S2). HPCs in the inflammatory microenvironment are not infected with HBV, which may be related with lack of HBV receptors. When HPCs are active within the liver streaming line, regenerated hepatocytes with HBV receptors may still have a chance to become infected with HBV (Figure 6).

At present, the exact role of HBV in the development of HCC remains complex and enigmatic, our study sheds light on an important role of HBsAg and its specific receptors and the implication of an inflammatory microenvironment. Our data collectively lends support to an important role of HBsAg and HBV receptors in the clinical outcome of HBsAg-positive HCC patients. The strong association of peritumoral HBsAg, NTCP and ASGPR expression with tumor recurrence suggests that these molecules could serve as useful biomarkers to stratify outcome and tumor recurrence risk. However, the basic researches about HBV and the related development of HCC have both been hindered by the lack of suitable *in vitro* infection systems and animal models, moreover, the inflammation microenvironment during the process of HCC formation is complicated and not invariable, which is difficult to demonstrate *in vitro*. If the effective infection systems and animal models are constructed, we would supply more experimental data to support our speculation. In conclusion, peritumoral expression of HBsAg and its specific receptors correlates with certain patient clinicopathologic characteristics and predicts survival and tumor recurrence in HBsAg-positive HCC patients.

## MATERIALS AND METHODS

### Patients and clinical data

Fresh and archived, formalin-fixed, paraffin-embedded liver tissue specimens were all acquired from HBsAg-positive patients with pathologically proven HCC who underwent curative resection at the Eastern Hepatobiliary Surgery Hospital between January 1997 and December 2007. HCC was staged according to the UICC TNM classification system (7^th^ Edition) and tumor differentiation was graded by the Edmondson-Steiner grading system. Acquisition of tissue specimens was approved by the Hospital Research Ethics Committee and performed in accordance with institutional and state guidelines on the use of human tissue specimens for experimental purposes.

### Follow-up

Patients were followed up by clinic visit every 2 months during the first postoperative year and at least every 3–4 months thereafter until study closure in October 2012 with serum AFP and abdominal ultrasonography. Progressive elevation of serum AFP levels and/or ultrasonographic detection of a new hepatic lesion prompted hospitalization for confirmation of diagnosis and appropriate management, including repeat resection, radiofrequency ablation (RFA), transcatheter arterial chemoembolization (TACE), or supportive therapy. Recurrence was confirmed by contrast-enhanced imaging studies according to standard guidelines for HCC as described previously [24]. OS was defined as the interval between the dates of surgery and death, whereas RFS was defined as the interval between the dates of surgery and recurrence. If recurrence was not diagnosed, patients were censored on the date of death or the last follow-up. Clinical follow-up was not disclosed to laboratory personnel until statistical analysis.

### Tissue microarray and immunohistochemistry

After hematoxylin and eosin (H&E)-stained slides were reviewed by three independent pathologists, we constructed slides for tumor microarray. Two cores were taken from each formalin-fixed, paraffin-embedded HCC and peritumoral tissue specimen, respectively, by using punch cores measured 1.5 mm in the greatest dimension from the non-necrotic area of the tumor and peritumoral tissue. Peritumoral tissue was 1.5 cm from the edge of the tumor. Standard heat-induced antigen retrieval was carried out. Immunohistochemistry was performed by a standard two-step method. The following primary antibodies were used: anti-HBsAg antibody (dilution 1:50; Invitrogen, Carlsbad, CA, USA), anti-NTCP antibody (dilution 1:40) and anti-ASGPR antibody (dilution 1:100) (both from Abcam, Cambridge, UK), anti-PCNA antibody (dilution 1:2000; Cell Signaling Technology, Danvers, MA, USA), anti-K7 antibody (dilution 1:50; Dako, Glostrup, Denmark), anti-CYP3A4 antibody (dilution 1:400; Sata Cruz Biotechnology, Santa Cruz, CA, USA) and anti-MRP2 antibody (dilution 1:200; Abcam). In negative controls, phosphate buffered saline (PBS) was used instead of the primary antibody. Cytoplasmic and membranous staining with yellow particles was considered positive under a Leica DMRA microscope (Leica Microsystems Imaging Solutions, Ltd., Cambridge, UK). For immunofluorescence microscopy, primary antibodies against the following proteins were used: HBsAg, NTCP, ASGPR and K7. Alexa488-conjugated goat anti-mouse (or anti-rabbit) IgG and Alexa568-conjugated goat anti-rabbit (or anti-mouse) IgG (all from Invitrogen) were used as secondary antibodies.

All sections with immunohistochemical staining were observed and the pictures of four representative fields were photographed by an Olympus microscope (IX-70 OLYMPUS, Japan) under high power view. The integrated optical density (IOD) in each image was measured with the same setting for all the slides, and the density was calculated as IOD/total area of each image. IOD > median in microarray tissue samples was considered high HBsAg, NTCP or ASGPR expression while IOD ≤ median was considered low HBsAg, NTCP or ASGPR expression. The proliferative index of ductular reaction (PI-DR) was calculated as the ratio of the number of PCNA immunoreactive nuclei to the total number of reactive ductular cells as described [11]. Specifically, four representative fields within each section were randomly chosen and captured under 200 x. The same fields were captured in consecutive sections and stained with anti-K7 antibodies for quantification of the number of reactive ductular cells in reactive ductules.

### Evaluation of necroinflammatory activity

Necroinflammatory activity was graded as described by Ishak *et al.* [25]. A score was assigned for each of interface hepatitis, confluent necrosis, focal lytic necrosis/apoptosis/focal inflammation and portal inflammation. A score of 1 to 4 represented no activity, 5–8 mild activity, 9–12 moderate activity, and 13–18 severe activity. Fibrosis was assessed as suggested by Desmet *et al*. [21] and categorized as absent or mild fibrosis (0), moderate or severe fibrosis (1–4), and cirrhosis (5–6).

### Fluorescence *in situ* hybridization (FISH)

Total DNA was extracted from 20 fresh HCC tissue specimens using Gentra Puregene tissue kits (Invitrogen) according to the manufacturer's instructions. HBV DNA was quantified according to a standard curve by fluorescence quantitative real-time polymerase chain reaction (PCR) using HBV PCR Diagnostic Kit (Daan Genetic Diagnosis Inc., Guangzhou, China) following the manufacturer's instruction. A HBV DNA FISH Kit (Haoyang Biotechnology, Tianjin, China) was used for dual color FISH. Tissue sections were deparaffined and dehydrated in gradient alcohol. After heat denaturation, hybridization solution containing full-length HBV DNA probes was added dropwise to paraffin sections. Then, they were conventionally incubated and washed at room temperature. Hybridization signals were detected by diaminobenzidine staining or fluorescence-conjugated antibody. The slices were washed with Tris buffered saline solution and observed by fluorescence microscopy.

### Statistical analysis

Statistical analysis was performed using SPSS 20.0 for Windows (SPSS Inc., Chicago, IL). Difference between paired-data was confirmed to be normally distributed and then analyzed by *T*-test, and differences between categorical variables were assessed by the Chi-square test or Fisher's exact test, when necessary. OS was defined as the interval between the dates of surgery and death, whereas RFS was defined as the interval between the dates of surgery and recurrence. If recurrence was not diagnosed during this study, patients were censored on the date of death or the last follow-up. Pearson's correlation coefficient was used to determine correlations between normally distributed continuous variables. Spearman's rank correlation test was employed to show rank-order correlations between the variables. Kaplan-Meier analysis was used to determine the survival. Log-rank test was used to compare patients' survival between subgroups. Differences were considered statistically significant for *p*-values less than 0.05 (two-tailed).

## SUPPLEMENTARY FIGURES AND TABLES



## Abbreviations

HBV: Hepatitis B virus
HCC: hepatocellular carcinoma
RFS: recurrence-free survival
HPCs: hepatic progenitor cells
NTCP: sodium taurocholate cotransporting polypeptide
ASGPR: asialoglycoprotein receptor
