# Supplementary material for: Hepatitis B virus (HBV) receptors: Deficiency in tumor results in scant HBV infection and overexpression in peritumor leads to higher recurrence risk
Source: Oncotarget. 2015 Oct 20;6(40):42952–62. doi: 10.18632/oncotarget.5518 (PMC4767483; doi:10.18632/oncotarget.5518)
Supplement: Supplementary file 1 [file oncotarget-06-42952-s001.pdf]

SUPPLEMENTARY FIGURES AND TABLES

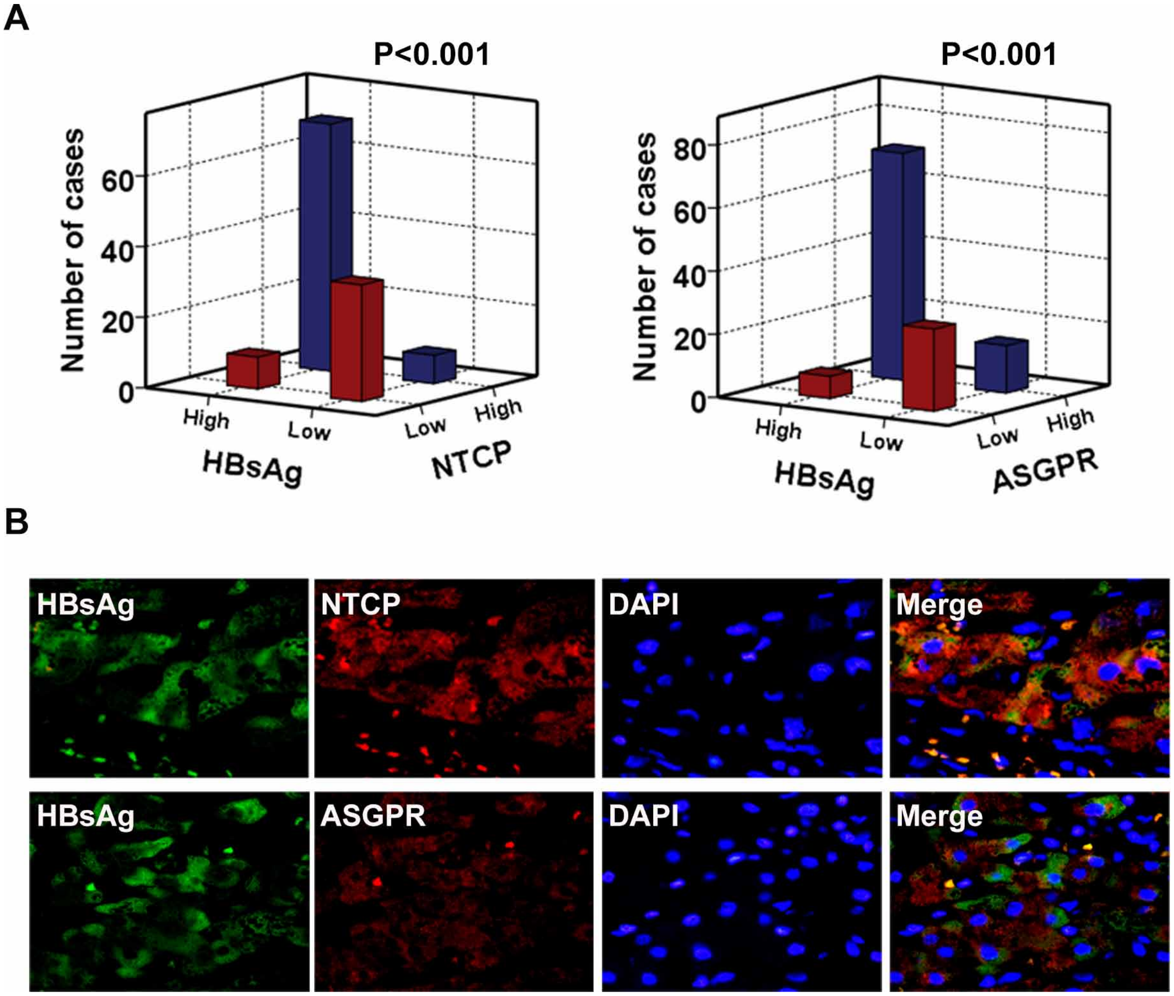

Supplementary Figure S1: The correlation between peritumoral expression of HBsAg and NTCP or ASGPR. A. The surrounding tumor tissues with different HBsAg expression have different levels of NTCP ($P < 0.001$) and ASGPR ($P < 0.001$). **B.** Some cases of peri-tumor with intensive HBsAg staining showing high expression of NTCP or ASGPR staining.

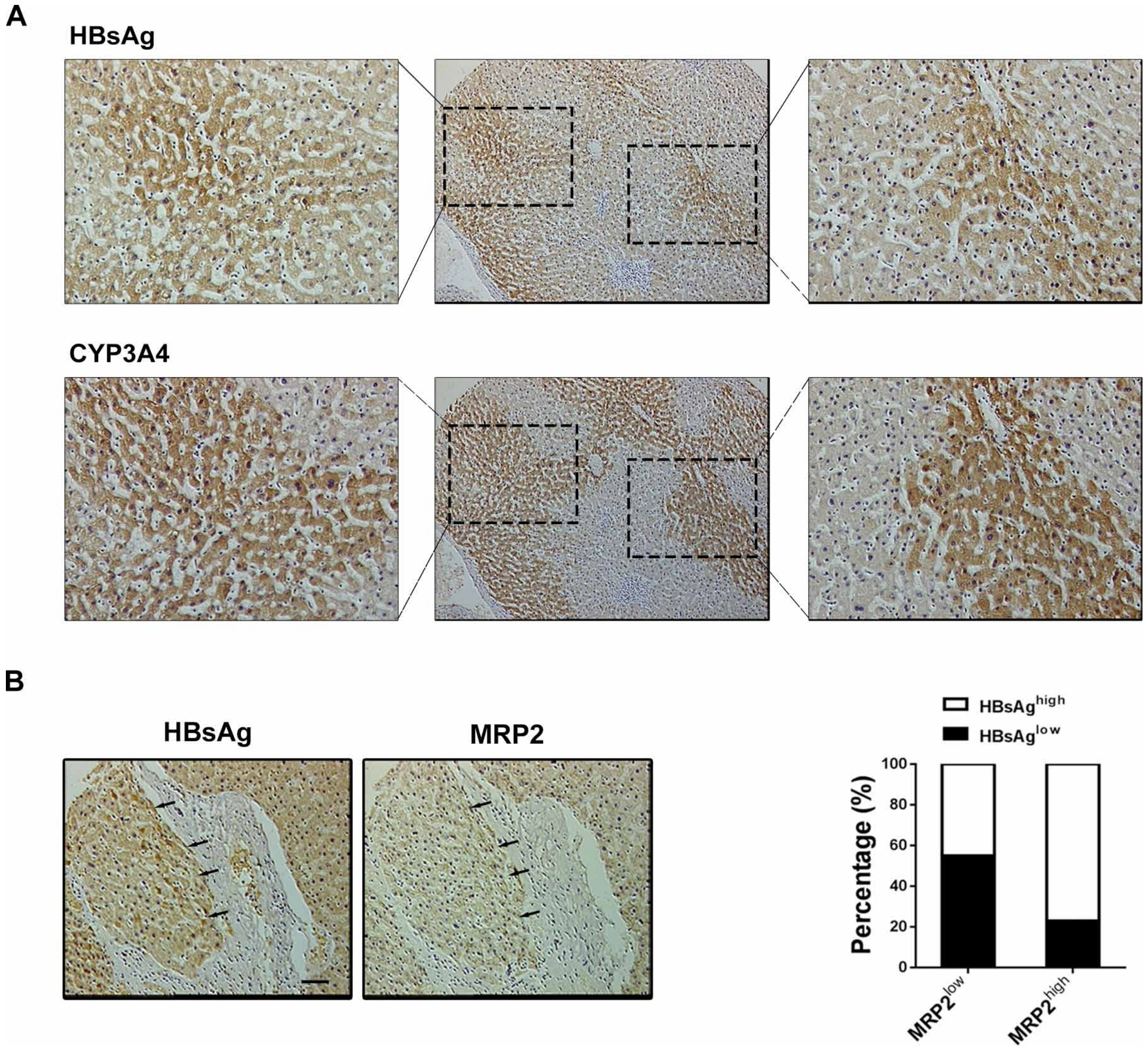

Supplementary Figure S2: Expression of HBsAg on mature hepatocytes. A. The immunostaining of CYP3A4, as a cytochrome enzyme exhibited by mature hepatocytes, showed a co-expression with HBsAg, which confirmed that HBV infection was mainly distributed in the central vein zone. **B.** The immunostaining showed that MRP2 expression, as a polarization marker of hepatocytes, was consistent with HBsAg expression.

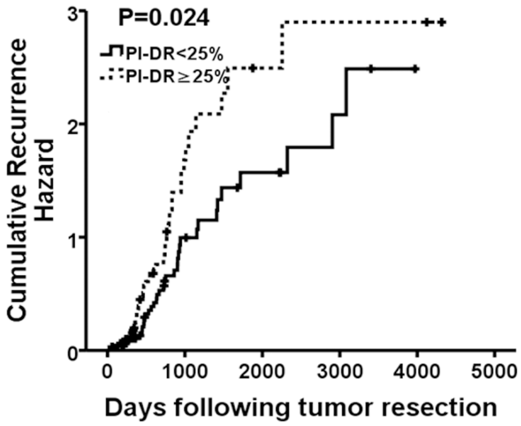

Supplementary Figure S3: Cumulative recurrence hazard analysis about PI-DR. Higher PI-DR could contribute to the risk of HCC recurrence ($P < 0.05$)

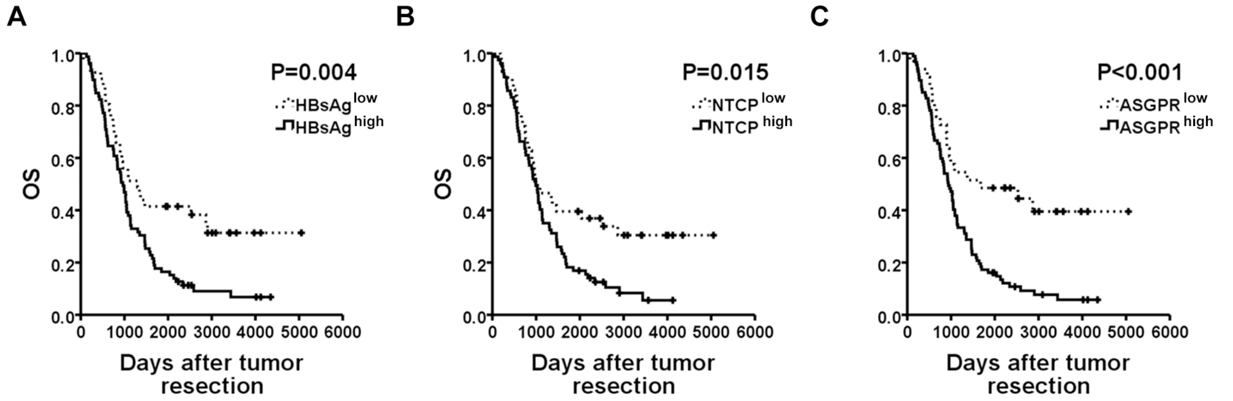

Supplementary Figure S4: Overexpression of peritumoral HBsAg, NTCP and ASGPR is associated with poorer OS. Survival analyses showed that patients with high peritumoral HBsAg were more likely to have shorter OS **A.** and with overexpression of NTCP or ASGPR were also associated with poorer prognosis **B.**

Supplementary Table S1: Patient demographic and baseline characteristics

Variables	Values	Percent
**Age (years)**	53.02 ± 11.62	
**Gender (Male/Female)**	97/22	80.8%/19.2%
**Tumor size (cm)**	5.9 ± 3.2	
**Lymph node metastasis**	10	8.3%
**Microvascular invasion**	69	57.5%
**Tumor encapsulation**	63	52.5%
**Edmondson grade**		
I, II	84	70%
III, IV	36	30%
**TNM**		
I, II	110	91.7%
III	10	8.3%
**α-Fetoprotein (ng/mL)**	335.2 ± 411.3 (1.7–1000.0)	
**Preoperative ALT (U/L)**	58.6 ± 38.5 (7.8–232.2)	
**Preoperative AST (U/L)**	41.2 ± 19.4 (2.3–118.3)	

Supplementary Table S2: Demographic and clinicopathologic features of HBsAg-positive hepatocellular carcinoma patients stratified by peritumoral expression of HBsAg, NTCP or ASGPR

Variables	Peritumoral HBsAg density		*P*	Peritumoral NTCP density		*P*	Peritumoral ASGPR density		*P*
	Low	High		Low	High		Low	High	
**Age (years)**									
< 60	31	63	0.602	32	62	0.437	25	69	0.673
**Male Gender**	33	64	0.945	36	61	0.548	27	70	0.866
**Tumor size (cm)**									
≥ 5	19	56	0.008	20	55	0.007	14	61	0.005
**Tumor encapsulation**									
Yes	20	43	0.557	21	42	0.548	15	48	0.341
**Microvascular invasion**									
Yes	7	62	< 0.001	13	56	< 0.001	9	60	< 0.001
**Edmondson grade**									
I, II	34	50	0.026	32	52	0.430	27	57	0.082
III, IV	7	29		11	25		6	30	
**TNM**									
I, II	37	73	0.734	39	71	0.744	31	79	0.725
III	4	6		4	6		2	8	
**AFP (ng/mL)**									
< 400	21	58	0.015	24	55	0.084	16	63	0.014
≥ 400	20	21		19	22		17	24	

Supplementary Table S3: Univariate analyses of factors associated with overall survival (OS) and disease free survival (RFS)

Factors	OS	RFS
**Age**: < 60 *vs*. ≥ 60 years	0.922	0.393
**Gender**: Male *vs*. Female	0.359	0.714
**Tumor size**: < 5 *vs*. ≥ 5 cm	0.002	0.005
**Tumor encapsulation**: no vs. yes	0.304	0.279
**Microvascular invasion**: no *vs*. yes	0.026	0.055
**Edmondson grade**: I, II *vs*. III, IV	0.203	0.424
**TNM**: I, II *vs*. III	0.171	0.342
**AFP**: < 400 *vs*. ≥ 400	0.869	0.777
**HBsAg expression**: Low *vs*. High	0.005	0.017
**NTCP expression**: Low *vs*. High	0.017	0.003
**ASGPR expression**: Low *vs*. High	0.001	0.002

Factors with $P < 0.100$ were selected for subsequent multivariate analysis.
